# The Feasibility and Acceptability of a Data Capture Methodology in Pediatric Cancer Patients Treated with Targeted Agents and Immunotherapies

**DOI:** 10.3390/curroncol31020051

**Published:** 2024-01-25

**Authors:** Karim Thomas Sadak, Taiwo Opeyemi Aremu, Seah Buttar, Daniel Van Ly, Brenda Weigel, Joseph P. Neglia

**Affiliations:** University of Minnesota Masonic Children’s Hospital, University of Minnesota Masonic Cancer Center, 420 Delaware St. SE—Mayo MMC 484, Minneapolis, MN 55455, USA; aremu006@umn.edu (T.O.A.); butta004@umn.edu (S.B.); lyxxx108@umn.edu (D.V.L.); weige007@umn.edu (B.W.); jneglia@umn.edu (J.P.N.)

**Keywords:** childhood cancer, survivor, consent discussion

## Abstract

As childhood cancer treatments have improved to include new and innovative agents, the need for more advanced monitoring of their long-term effects and related research has increased. This has resulted in a need for evidence-based research methodologies for the longitudinal care of childhood cancer patients treated with targeted agents and immunotherapies. The rationale for this pilot study was to determine the feasibility and acceptability of a data capture methodology for pediatric, adolescent, and young adult cancer patients treated with targeted agents and immunotherapy as there is little research to inform this delivery of care. Data were collected from thirty-two patients and two providers for descriptive statistics and thematic analyses. Feasibility was characterized by expected participant attrition. Key drivers of acceptability were (1) providers’ language and clarity of communication and (2) convenient participation requirements. Long-term follow-up research practices developed with input from key stakeholders, including patients, caregivers, and providers, can lead to acceptable and feasible research protocols that optimize successful participant recruitment. These evidence-based research practices can result in high participant satisfaction and can be implemented as program development initiatives across centers caring for childhood cancer survivors.

## 1. Introduction

Advancements in the treatment of pediatric/adolescent cancers have resulted in survival rates as high as 85% [1,2]. With these advancements comes the need to monitor the long-term effects of treatment, including subsequent neoplasms, decreased fertility, and cardiac outcomes [3,4]. As many as 62.3% of survivors report one or more chronic health conditions, with 27.5% reporting severe conditions [5]. Robust models of care with evolving evidence-based practice guidelines have been designed and implemented to meet the needs of this childhood cancer survivor population and the new treatments they are receiving.

Targeted therapies and immunotherapies are being used more frequently for a variety of childhood cancers. With the advent of these newer therapies, more cancer patients are surviving into adulthood [6]. While increased survival remains the primary goal with most novel agents and new treatments, additional considerations now more than ever include improving long-term patient wellbeing and reducing late effects of treatment.

Research focused on the late effects of targeted agents and immunotherapies is necessary to identify their long-term risks and inform practice guidelines for necessary follow-up care. Previous literature on this topic has primarily analyzed retrospective cohorts to assess chronic health conditions that provided limited exposure data to targeted agents and immunotherapies [5].

There is a growing need for additional research on the late effects of cancer treatment with targeted agents and immunotherapies to allow earlier surveillance and therapeutic interventions to decrease the risk of related late effects. One challenge for such research is that this patient population is highly mobile, particularly during the transition into young adulthood, where survivors experience educational, employment, or personal life changes [7]. There is limited literature on the acceptability and feasibility of longitudinal follow-up for childhood cancer survivors that specifically have received targeted agents and immunotherapies. The primary objective of this pilot study was to develop and test the feasibility and acceptability of a generally accepted data capture methodology for longitudinal clinical surveillance in pediatric/adolescent/young adult cancer patients specifically treated with targeted agents and immunotherapies [8,9].

## 2. Methods

### 2.1. Study Design

**Figure 1:**
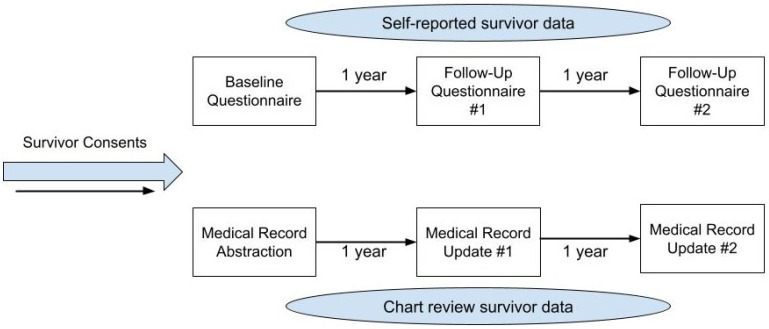
Description of data capture methodology with timeline.

An exploratory prospective cohort pilot study was designed using a mixed-methods approach to assess the feasibility and acceptability of the proposed data capture methodology (Figure 1).

The development of the data capture methodology was based on the current research infrastructure of a childhood cancer survivor program (CCSP), which includes a combination of longitudinal data self-reported by survivors and longitudinal data abstracted from survivors’ medical records (Figure 1). This model provided the context for the feasibility and acceptability assessments.

Feasibility was defined as the number of patients that were successfully recruited for enrollment and the number of patients that successfully completed the 1- and 2-year follow-up. Acceptability was defined as (1) patient or caregiver satisfaction throughout the study, including the recruitment/enrollment process, (2) provider satisfaction with the recruitment/enrollment process, and (3) key informant interviews after the recruitment/enrollment process (patient/caregiver/provider).

### 2.2. Participant Recruitment

Following the Institutional Review Board (IRB) approval of this study (IRB ID: 1611M00621), patients less than or equal to 29 years of age (or their families) receiving care in a Division of Pediatric Hematology/Oncology were recruited for this study following their informed consent. All such patients received care in a pediatric oncology clinic that delivers pediatric- and adult-centered care to survivors of all ages. The institutional practice is to typically transfer to adult care settings when survivors are 30 years of age. Thus, eligible patients for this study were captured over a 16-month period through all routine survivor-focused care as the clinic remained within the Division of Pediatric Hematology/Oncology. Eligible patients included those who had received or were planning to receive at least one targeted agent or treatment that targets the immune system, including immuno-oncology agents. These were broadly defined to include as many eligible survivors as possible. The same rationale was employed for including both patients off therapy and those that were receiving immunotherapies as part of their upcoming treatments. Patients under the age of 18 had parents that participated. Non-English-speaking patients as well as patients with less than or equal to six months’ life expectancy were excluded from participating in this study. The same questionnaire was required to be completed as baseline questionnaire, follow-up questionnaire #1, and follow-up questionnaire #2 (Table S1).

### 2.3. Data Collection

Following the patients’ consent, demographic information (including parents’ and/or caregivers’ name, address, and contact information for pediatric patients), family history, primary diagnosis history, secondary diagnoses, and previous treatment history (including chemotherapy drugs and cumulative doses, radiation therapy fields and doses, and surgical procedures) were collected through medical record abstraction.

Following this initial medical record abstraction, data were collected with the use of questionnaires and in-depth semi-structured interviews. The baseline questionnaire was completed by the patients/families, detailing self-reported health conditions and a review of systems, including psychosocial risk factors. The follow-up questionnaires were mailed to the respondents’ physical address, with a self-addressed stamped return envelope every 12 months for two years. Participants also could return the questionnaire in person during their routine survivor-focused care. Follow-up phone calls were made once a week for 3 weeks when questionnaires were not returned within one month of being sent out. Medical record updates were also completed every 12 months for two years to better understand longitudinal participation by patients and collect longitudinal data on therapy-related toxicities. This was also used to validate the self-reported data on the follow-up questionnaires. Interviews were conducted via recorded phone calls.

The feasibility of the data capture methodology was assessed by evaluating longitudinal participation.

The acceptability of the proposed data capture methodology was assessed using both quantitative (survey) and qualitative (phone interviews) data. Data that assessed the satisfaction of all key stakeholders (patients/parents/providers) were collected at the time of the consent discussion using an institutional healthcare satisfaction survey [8]. During the phone interviews, participants were asked about their perceptions about the study, their motivations, as well as potential barriers to participating in the study.

The accrual goal of 20–30 patients was deemed sufficient to achieve the primary objective of this exploratory pilot study, which included qualitative data collection per standard phenomenological qualitative study requirements to achieve informational redundancy and content saturation [10,11]. The study design did not include a control group as it was not powered to detect significant changes because of the interventions. Instead, the goal was to evaluate the feasibility and acceptability of a data capture methodology.

### 2.4. Analysis

Descriptive statistics were used to summarize the survey data assessing feasibility and acceptability. Using N5Vivo 9.0 to read, organize, and code the interview transcripts, multiple authors (K.T.S., T.O.A., and S.B.) identified major themes in the interviews used for acceptability assessments through thematic analysis using deductive approaches [12,13]. Initial codes were identified during discussions between researchers and a review of the most relevant literature. The coding researchers met at defined intervals to discuss the evolving themes within the predetermined categories, re-coding, the associated quotes, and their interpretations.

## 3. Results

A total of thirty-two patients and two providers participated in this study. All patients were currently receiving medical care in a single institution (CCSP clinic) within a single large academic health center in the Upper Midwest region of the United States. Participant ages ranged from 8 to 27 years. All patients but two had completed treatment, with varying periods of time off therapy, and were actively receiving medical long-term follow-up care at this single institution. Of the 32 enrolled participants, 50% (*n* = 16) completed the phone interview; and of the 16 that completed the interview, 43.8% (*n* = 7) were parents of survivors, and 56.3% (*n* = 9) were patients (survivors). Demographics of participants that completed the interview are summarized in Table 1.

### 3.1. Feasibility (Participation Data)

**Figure 2:**
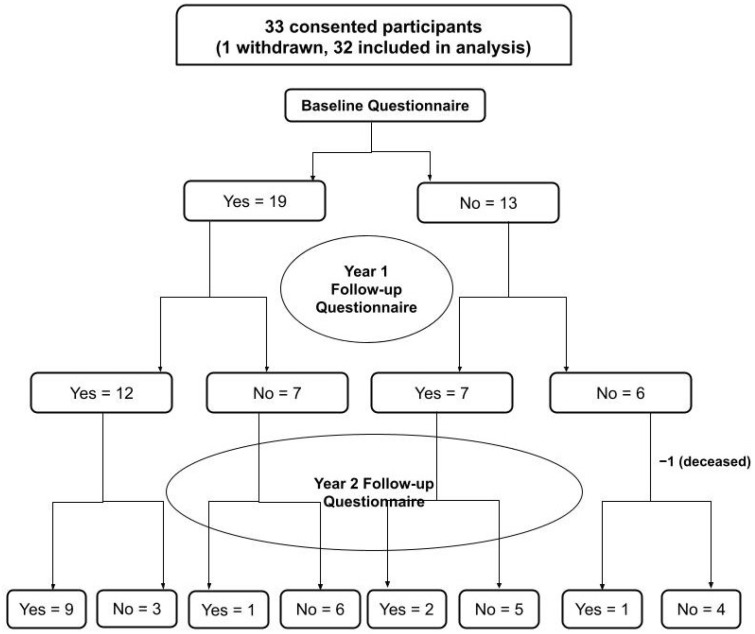
Flowchart summarizing subject participation at each study stage.

A convenience sample of 33 patients/families were approached about this study and completed the consent discussion. Of the 33 patents/families approached, 32 (97.0%) patients were successfully recruited for enrollment. Of the 32 patients/families enrolled, 19 (59.4%) completed and returned the follow-up questionnaire #1 at 1-year follow-up, while 13 (40.6%) of the 32 enrolled patients completed and also returned the follow-up questionnaire #2 at 2-year follow-up [Figure 2].

### 3.2. Acceptability (Survey Data)

Of the 33 patients that completed the consent discussion, 27 (81.8%) completed the satisfaction survey. Participants were asked if they strongly agree, somewhat agree, are uncertain, somewhat disagree, and strongly disagree with the following statements: (1) My provider listened carefully to what I said, (2) My provider explained things in a way that was easy to understand, (3) I received the information I needed from my provider, (4) My provider helped me feel like a partner in care, (5) I was able to share all the necessary information with my provider, (6) My provider spent enough time with me, (7) My provider answered all my questions. All 27 respondents selected “strongly agree” to all the questions, except two respondents that selected “somewhat agree” to “My provider helped me feel like a partner in care”.

### 3.3. Acceptability (Interview Data)

Participants were asked to provide their perceptions around the study, their motivations, as well as potential barriers to participating. From this information, two prominent themes emerged: (1) provider language and clarity of communication determined the acceptability of the consent discussion, and (2) optimizing convenience for participants was most important when considering the acceptability of the consent discussion.

#### 3.3.1. Theme 1: Provider Language and Clarity of Communication Determined the Acceptability of the Consent Discussion

Participants reported that the providers’ approach and clarity of communication were essential to acceptance during consent discussions. This included the use of specific language and keywords that brought clarity to the discussion. Respondents felt that a key message was to clearly illustrate how participation would improve outcomes for future children with cancer. Privacy concerns were also raised by participants and, therefore, specific language to address the protection of personal health information, as is required under the HIPAA law, was also identified as an important factor in making the consent discussions more acceptable. Thinking about their consent discussions, participants raised the concern of potential future study visits and related location and procedures associated with participation. Clear and specific language detailing these requirements was also reported to be a critical component of acceptability for the participants.

1. Subtheme 1a: Providers clearly communicating that research participation could help other childhood cancer survivors was a key driver of acceptability.

Participants were motivated by how clearly the providers were able to highlight the importance of this research. This fueled a desire to help those who may be impacted by similar health challenges in the future. Participants had a shared sentiment, stressed by their willingness to do anything possible to help present and future patients. Having experienced cancer treatments that included novel agents and/or immunotherapies, participants were willing to participate in research to help ensure that others with similar health conditions do not have the same experiences (see comments from participants 9 and 20 in Subtheme 1a of Table 2).

2. Subtheme 1b: Providers clearly communicating strategies to protect participant privacy was important for acceptability.

Participants reported that when providers clearly communicated the importance of maintaining their privacy, this built trust and supported the acceptability of the consent discussion (see comments from participants 5 and 14 in Subtheme 1b of Table 2).

3. Subtheme 1c: Providers using clear language repeatedly highlighting the voluntary nature of participation was supportive of acceptability.

Participants were exceedingly appreciative of the interviewers’ time, patience, and demeanor (see comments from participants 14 and 22 in Subtheme 1c of Table 2).

The provider’s ability to clearly emphasize the voluntariness in participation gave the participants a sense of reference to their liberty and freedom of choice, hence not feeling coerced by their provider to participate.

#### 3.3.2. Theme 2: Optimizing Convenience for Participants Was Most Important When Considering the Acceptability of the Consent Discussion

Participants reported that it was important for providers to clearly communicate the potential scheduling implications of research participation. For participants, it was critical to have a clear understanding of how convenient or inconvenient participation would be, including study visits, ancillary testing, or any other aspect of participation. Also, having the consent discussion in a familiar care setting promoted optimal convenience and comfort for participants, thus supporting acceptability.

1. Subtheme 2a: Having consent discussions in familiar spaces and places promoted acceptability for participants.

The ability to have the consent discussion about participating in research in a familiar location, such as where their routine follow-up care is delivered, was seen as highly favorable by the participants (see comments from participants 2, 5, and 8 in Subtheme 2a of Table 2).

2. Subtheme 2b: Providers clearly communicating what clinic visits and additional tests were needed through research participation was key in their decision-making process.

The consensus among those interviewed was that having no research-only testing did make their participation more likely and the consent discussion more comfortable. All participants reported it was important to understand the subsequent time commitment prior to agreeing to participate but many also indicated that this was secondary in importance to understanding how their participation and the research outcomes in general would help others (see comments from participants 7 and 28 in Subtheme 2b of Table 2). 

### 3.4. Providers

Two of the three eligible providers completed all of the consent discussions. These two providers provide over 90% of patient care to childhood cancer survivors. They were interviewed on their experience with the participants while conducting the consent discussion. The recurrent themes identified here included overlap with those from the research participants. Both providers reported noticing a deep sense of gratitude from participants and this prioritized the importance of this research impacting others as a critical component of acceptability to participants:

*“My impression at least was they all felt like this was an opportunity to give back.”*

(Provider 1)

This provider went on to say,

*“The childhood cancer survivor almost uniformly had verbalized a sense of gratitude for being where they’re at, for having gotten through what they went through, having it so far in their past, that they all seemed happy to help through participating in research.”*

(Provider 1)

Optimizing the participant experience was also highlighted by providers:

*“The study was pretty smooth. It was a very easy, straightforward consent from my end. I don’t think it added to anybody’s stress.”*

(Provider 2)

The convenience of participation was highlighted by a provider when describing the consent discussion:

*“It was much easier, a much lower stakes conversation. Almost uniformly in that population they were all willing and happy to participate.”*

(Provider 1)

## 4. Discussion/Conclusions

Data capture methodologies are integral to improving long-term outcomes in pediatric/adolescent cancer survivors. Ensuring the acceptability and feasibility of such data capture methodologies will support long-term follow-up studies. This is most needed in survivor-focused care after treatments with targeted agents and immunotherapies, as there are minimal practice guidelines. With sound clinical research infrastructure, descriptive research can effectively identify the relevant late-effects risks so the necessary clinical surveillance can be implemented for survivors. Providers, researchers, and health system leaders must all pay close attention to the drivers of acceptability for patient research participation so that learning health systems can be built that promote feasible longitudinal research participation by engaged patients and ultimately leads to rapid cycle improvements in clinical and research practices. Implementation challenges may be patient- or provider-centered and often exist at the level of the health system. For survivors of childhood cancer treated with targeted agents and immunotherapies, the acceptability of a proposed data capture methodology is hinged on the providers’ approach and ability to communicate clearly during the consent process and the convenience of participating in this long-term follow-up study. The electronic health record (EHR) system has been instrumental in providing convenience and easy access, anchored on patient-centered care, as avenues for improving health outcomes [14].

The concept of participant convenience served as a primary facilitator of acceptability. The physical location of consent discussions was highly supportive of a positive experience for participants when the discussions took place in familiar clinic spaces. Participants reported a sense of comfort and greater willingness to participate in the proposed research when considering it in a familiar setting. It was no surprise that convenience was further described by participants to be specifically related to the number of additional clinic visits needed and the number and types of follow-up testing included in the research.

The identified themes of acceptability are similar to those seen in adherence and compliance studies with care recommendations. The impact of easily understood and clear communication, from providers of all disciplines, has been associated with enhanced acceptance and adherence to long-term therapies and preventative care [15,16,17]. Some survivors described that their acceptance of the consent discussion directly hinged on the provider’s ability to answer their clarifying questions. This emphasized the importance of active listening, which is essential to optimal provider–patient relationships [18] and would seemingly extend to discussions of research participation. This study addresses a potential gap in the literature by providing feasibility and acceptability data that can guide practices for long-term follow-up studies in the population of childhood cancer survivors treated with targeted agents and immunotherapies. For example, having related consent discussions in familiar clinic locations and then imbedding longitudinal data collection into routine follow-up care was described as highly desirable by participants. However, this familiarity with care settings can be disrupted when an AYA-aged patient transitions from pediatric- to adult-centered care [19]. This emphasizes the need for coordinated and deliberate transition protocols for childhood cancer survivors to help support continued participation in late-effects research. In addition to the challenges of potential research participants that are transitioning care, there is a critical need to support inclusion and equity as core principles in all research efforts. Researchers must consider all aspects of acceptability to help overcome patient mistrust of research and the health system in general [20]. Inclusive research practices must include insight from and considerations of all key stakeholders and additional work is necessary to examine the feasibility, acceptability, and participant satisfaction of similar long-term follow-up research, both for childhood cancer survivors and other populations of patients with chronic diseases of childhood.

Limitations to this study include the presence of selection bias, as most participants were already receiving routine long-term follow-up care. Satisfaction survey data and interview data on acceptability were subject to participant recall bias as some interviews happened several days or weeks after the encounter with the medical provider. The single-institution patient cohort also limits the generalizability of the findings to different populations of childhood cancer survivors. A longer duration of follow-up in this study would have helped confirm that patients not completing year 2 questionnaire were in fact truly lost to follow-up. It is unclear if these participants would have completed follow-up questionnaires in year 3 or 4, for example.

The future direction of this work would include the implementation of similar research protocols for longitudinal follow-up in health systems of varied sizes and resources. A larger cohort of eligible participants would provide increased power to support the findings from this study’s quantitative data and develop stop/go criteria for future pilot work, if needed. Similar study designs could be embedded in early-phase trials of new targeted agents and immunotherapies as this work focused on long-term childhood cancer survivors that had previously received such agents. Downstream outcomes beyond participant satisfaction can be studied by as well as program development initiatives across a diverse group of centers caring for childhood cancer survivors.

## Figures and Tables

**Table 1:** Summary table describing patient/parent participants that completed the interview.

Participant #	Survivor Gender	Survivor Age (Years)	Participants	Survivor Diagnosis	Targeted Agent and/or Immunotherapy Received
1	F	14	Parent	Osteosarcoma	Olaparib
2	F	10	Parent	Alpha thalassemia major	ATG, alemtuzumab
4	M	9	Parent	ALL	Bortezomib
5	M	19	Patient	Neuroblastoma	IL-2, dinutuximab
6	M	20	Patient	Osteosarcoma	Dinutuximab
7	M	9	Parent	Neuroblastoma	Dinutuximab, rituximab
8	M	19	Patient	Diffuse large B cell lymphoma	Rituximab
9	M	16	Patient	Aplastic anemia	ATG
14	M	8	Parent	Neuroblastoma	IL-2, dinutuximab
15	F	27	Patient	CML	Imatinib
16	M	19	Patient	Medulloblastoma	Cis-retinoic acid
17	M	26	Patient	CML	Imatinib
20	M	27	Patient	ALL	Imatinib
22	F	26	Patient	Neuroblastoma	Cis-retinoic acid
28	M	14	Parent	Diamond–Blackfan anemia	ATG
29	M	13	Parent	Burkitt’s lymphoma	Rituximab

ALL: Acute lymphoblastic leukemia. CML: Chronic myeloid leukemia. ATG: Anti-thymocyte globulin.

**Table 2:** Summary of key quotes from identified themes/subthemes from patient/parent interviews on satisfaction of consent discussions.

Theme/Subtheme	Key Quotes
1. Provider language and clarity of communication determined the acceptability of the consent discussion.	
1a. Providers clearly communicating that research participation could help other childhood cancer survivors was a key driver of acceptability.	*Like I said, I like doing it [research participation] and I would like to do stuff like this just so that other people like me that come up don’t have to go through what I went through. (Participant 9)* *I’m always willing to participate and if I can do anything to help further research, that’s what I’m here for. I’ve been through stuff and if I can help people in the future going forward, I’m all for it. (Participant 20)*
1b. Providers clearly communicating strategies to protect participant privacy was important for acceptability.	*I always have concerns about privacy, but nothing serious about this study after she explained things to me, and I thought that was handled with the patience that she had in introducing the study. (Participant 14–parent of survivor)* *He explained all the privacy stuff, and yeah, he went into good detail, he explained it all, and I’m not really worried about anything like that. I’m more worried about my internet privacy than that. (Participant 5)*
1c. Providers using clear language repeatedly highlighting the voluntary nature of participation was supportive of acceptability.	*“I felt like I was getting a really, really careful conversation and discussion, and I just thought she did a great job” (Participant 14–parent of survivor).* *“I have nothing but the best things to say. He presented it kindly and with zero pressure. (He mentioned that) I did have this treatment and if I would want to participate in this study, it was totally up to me. I thought it was professional. I thought it was low pressure and kind.” (Participant 22)*
2. Optimizing convenience for participants was most important when considering the acceptability of the consent discussion.	
2a. Having consent discussions in familiar spaces and places promoted acceptability for participants.	*The place [clinic] where it was presented was absolutely fine … throughout the journey, that place has always been where the journey started, and it is eventually going to help us out. So, I thought that was a good place to start the conversation. (Participant 2- parent of survivor)* *No, it [the location of the consent conversation] was all good. I just went down to my appointment, and we were in the room that he did my yearly follow-up in, and he was like, ‘Oh, yeah. By the way, we also have this study.’ (Participant 5)* *He was just presenting me with the sheets and going over, individually, the pages about the study and then giving me more background information on it, and I think being there helped in terms of, like, I remember going there [in the clinic] to get chemotherapy, and now I’m on the other side and I’m fine. So I think that was really powerful. I mean, that’s just me personally, but I think that could also affect other survivors. (Participant 8)*
2b. Providers clearly communicating what clinic visits and additional tests were needed through research participation was key in their decision-making process.	*I think that’s great. He’s had enough tests and blood draws in his life, so the fewer, the better. (Participant 7–parent of survivor)* *It definitely matters that we weren’t being asked to visit a lab or have additional blood draws or any additional medical intervention, outside of what would normally be needed for his care. That’s not easy for us to do. We’re four hours away. (Participant 28–parent of survivor)*

## Data Availability

The data presented in this study are available on request from the corresponding author. The data are not publicly available due to privacy reasons.

## Supplementary Materials

The following supporting information can be downloaded at: https://www.mdpi.com/article/10.3390/curroncol31020051/s1, Table S1: Long-term follow-up clinic questionnaire.
