# Supplementary material for: The Feasibility and Acceptability of a Data Capture Methodology in Pediatric Cancer Patients Treated with Targeted Agents and Immunotherapies
Source: Curr Oncol. 2024 Jan 25;31(2):693–703. doi: 10.3390/curroncol31020051 (PMC10887547; doi:10.3390/curroncol31020051)
Supplement: Supplementary file 1 [file curroncol-31-00051-s001.zip › curroncol-2787201-supplementary.pdf]

LONG-TERM FOLLOW-UP CLINIC QUESTIONNAIRE

Appointment date: ______________________

Current Home Address and Contact Information of Patient

Name: ______________________

Birth date: ______________________

Street: ______________________

City/State/Zip: ______________________

Home phone: ______________________

Work phone: ______________________

Cell phone: ______________________

E-mail: ______________________

Patient's Primary Care Physician

Name: ______________________

Clinic: ______________________

Street: ______________________

City/State/Zip: ______________________

Phone: ______________________

Race/Ethnicity

Race: ______________________

Are you Hispanic? ☐ Yes ☐ No

Immunizations up to date? ☐ Yes ☐ No

Specialty Physician(s) (For example, Endocrinologist)

1. Name: ______________________
   Specialty: ______________________
   Clinic: ______________________
   Street: ______________________
   City/State/Zip: ______________________
   Phone: ______________________
2. Name: ______________________
   Specialty: ______________________
   Clinic: ______________________
   Street: ______________________
   City/State/Zip: ______________________
   Phone: ______________________
3. Name: ______________________
   Specialty: ______________________
   Clinic: ______________________
   Street: ______________________
   City/State/Zip: ______________________
   Phone: ______________________
4. Name: ______________________
   Specialty: ______________________
   Clinic: ______________________
   Street: ______________________
   City/State/Zip: ______________________
   Phone: ______________________

Patient Cancer Treatment History

1. Do you know what type of cancer you had?
   ☐ Yes ☐ No ***If yes***, what was it? ______________________

2. Do you know what type of treatment you received (i.e. surgery, chemotherapy, radiation)? ☐ Yes ☐ No

   If yes, What type of surgery did you receive? ______________________

   What type of chemotherapy did you receive? ______________________

   Did you receive radiation? ☐ Yes ☐ No

   If you received radiation, Do you know what body areas were irradiated and the dosages? ☐ Yes ☐ No

   ⇒ Please list areas of radiation and dosages, if known: ______________________

   ____

4. Are you aware of any late effects that you have from your cancer treatment? ☐ Yes ☐ No

   If yes, What are they? ______________________

5. What additional information would you like regarding late effects from your cancer treatment? ______________________

   ____

SOCIAL HISTORY

Marital Status and Living Arrangements

1. Marital status: ________________________________

2. Are you currently working?

   ☐ Yes ☐ No ☐ Does not apply

   If yes,

   What is your occupation?

   ____

   If no,

   Were you working before diagnosis of cancer?

   ☐ Yes ☐ No ☐ Does not apply

   Are you retired?

   ☐ Yes ☐ No ☐ Does not apply

   Are you disabled?

   ☐ Yes ☐ No

3. What is your living situation (for example, single family home, apartment, dorm, group home, etc)?

   ____

4. Who do you live with?

   ____

5. Do you feel that you have adequate social support and interaction?

   ☐ Yes ☐ No

Health Insurance

Since diagnosis of cancer have you had problems with obtaining or updating health insurance?

☐ Yes ☐ No

School History

1. Are you currently enrolled in school? ☐ Yes ☐ No

   If yes,

   Indicate grade level: _____ What is your grade average: _____

2. Indicate the highest grade **completed** in school:

   ☐ 1 - 8 years (grade school)
   ☐ 9 - 12 years (high school); did not graduate
   ☐ Graduated from high school
   ☐ Did not graduate, but obtained GED certificate
   ☐ Completed nongraded special education program
   ☐ Training after high school, other than college
   ☐ Some college; did not graduate
   ☐ Graduated from college; year of graduation
   ☐ Postgraduate education

<u>If not currently enrolled, continue on to next page. If you are currently in school (K-12), proceed with the next questions.</u>

3. Have you had any of the following in school:

Tutoring?	☐ Yes	☐ No
Resource/special education services?	☐ Yes	☐ No
Need to repeat grade(s)?	☐ Yes	☐ No
Behavioral problems affecting school performance?	☐ Yes	☐ No

4. Do you currently have an IEP (individualized education plan) in place?

   ☐ Yes ☐ No

5. Do you currently have a 504 plan in place?

   ☐ Yes ☐ No

6. Are you satisfied with the services you are receiving in or outside of the school setting?

   ☐ Yes ☐ No

7. Indicate any other kinds of help you are receiving in or outside of the school setting:

Speech/Language	☐ Yes	☐ No
Occupational Therapy	☐ Yes	☐ No
Physical Therapy	☐ Yes	☐ No
Counseling	☐ Yes	☐ No

HEALTH HABITS

Tobacco

1. Do you currently smoke cigarettes?

   ☐ Yes ☐ No

   If yes,

   How many years have you been smoking? ___________

   How many cigarettes per day? ___________________

2. Have you smoked cigarettes in the past?

   ☐ Yes ☐ No

   If yes,

   How many years did you smoke? _________________

   How many cigarettes per day? ___________________

Alcohol

1. Do you drink alcohol?

   ☐ Yes ☐ No

   If yes,

   How many drinks per week?

   ☐ < 1 per week

   ☐ 1-3 per week

   ☐ 4-7 per week

   ☐ > 7 per week

   On average, how many drinks per occasion?

   ☐ 1

   ☐ 2-3

   ☐ More than 3

Other Recreational Drugs

1. Do you currently use other recreational drugs? (i.e. marijuana, heroin, meth, cocaine etc)

   ☐ Yes ☐ No

2. Have you used other recreational drugs in the past?

   ☐ Yes ☐ No ☐ Does not apply

   If yes, for how many years? ____________________

3. Have you ever had treatment for alcohol or drug dependency?

   ☐ Yes ☐ No ☐ Does not apply

Exercise/Physical Activity

1. Do you participate in routine exercise or physical activity?

   ☐ Yes ☐ No

   If yes,

   What types of exercise do you perform?

   ____

   How many minutes do you exercise? ________________

   How many times per week do you exercise? ___________

   If no,

   Did you participate in routine exercise or physical activity before cancer diagnosis/treatment?

   ☐ Yes ☐ No

SEXUAL/REPRODUCTIVE HEALTH

☐ ***Check if the following questions do not apply to you***

1. Are you currently sexually active? ☐ Yes ☐ No

2. Have you:

   Conceived one or more children *before* cancer/BMT? ☐ Yes ☐ No

   Conceived one or more children *after* cancer/BMT? ☐ Yes ☐ No

   Adopted one or more children *after* cancer/BMT? ☐ Yes ☐ No

3. Do you have future plans to conceive? ☐ Yes ☐ No

4. Were you offered any method for pre-cancer preservation of fertility (i.e sperm banking, embryo cryopreservation)? ☐ Yes ☐ No

   If yes,

   Did you take advantage of this? ☐ Yes ☐ No

   If yes,

   What method was used? ______________________________

FOR WOMEN

☐ ***Check if none of the following questions apply to you***

1. Have you ever had a menstrual period? ☐ Yes ☐ No

   If yes,

   Age at your first period: ______________________________

   Last menstrual period (month/year): ____________________

2. Have you been pregnant? ☐ Yes ☐ No

   If yes,

   Number of pregnancies: ______________________________

   Number of deliveries: ______________________________

   Age at first delivery: ______________________________

3. Have you gone through menopause? ☐ Yes ☐ No

   If yes,

   Age at menopause: ______________________________

4. Have you ever taken birth control pills? ☐ Yes ☐ No

   If yes,

   Age started: __________ Age stopped: ______________

5. Have you ever taken estrogen/hormone replacement therapy? ☐ Yes ☐ No

   If yes,

   For how long? ______________________________

FAMILY MEDICAL HISTORY

Please list below any of your close relatives who have or had a serious medical condition, including ***cancer, tumors, leukemia, heart disease, high cholesterol requiring medication, high blood pressure requiring medication, stroke, diabetes or any other serious condition***. Indicate the side of your family the relative belongs to if applicable, for example, “maternal grandfather” or “paternal aunt.”

Relationship to you (include side of family, if applicable)	Specific condition this person had	Age at onset

CURRENT MEDICAL CONDITIONS

Check all that currently apply:

General

- ☐ Overweight ☐ Underweight
- ☐ Weight gain ☐ Weight loss

Skin

- ☐ Skin problems ☐ Tumors or moles ☐ Skin biopsies
- ☐ Other: ______________________________

Head/Eyes/Ear/Nose/Throat/Mouth/Neck

- ☐ Head injury ☐ Migraine or chronic headaches
- ☐ Stroke or TIA ☐ Earaches
- ☐ Mouth problems ☐ Nasal problems
- ☐ Chronic sore throat ☐ Blindness
- ☐ Cataracts ☐ Frequent nose bleeds
- ☐ Hearing loss ☐ Throat problems
- ☐ Glaucoma ☐ Other: ______________________

Pulmonary (Lungs)

- ☐ Asthma ☐ Chronic bronchitis
- ☐ Emphysema ☐ Pulmonary embolus
- ☐ Shortness of breath ☐ Chronic cough
- ☐ Other: ____

Cardiovascular (Heart)

- ☐ Angina (chest pain) ☐ Heart attack
- ☐ Heart infection ☐ Heart murmur/irregular beat
- ☐ High blood pressure ☐ High cholesterol
- ☐ Heart failure ☐ Fluid around heart
- ☐ Raynaud's phenomenon
- ☐ Other: ____

Hematologic

- ☐ Anemia ☐ Bleeding disorder
- ☐ Iron overload
- ☐ Other: ____

Immune/Infections

- ☐ Graft versus host disease
- ☐ HIV ☐ Tuberculosis
- ☐ Splenectomy ☐ Rheumatoid arthritis
- ☐ Lupus ☐ Chronic fatigue syndrome
- ☐ Other: ____

Psychiatric/Psychosocial

- ☐ Sleep problems ☐ Bipolar disorder
- ☐ Depression ☐ Panic attacks
- ☐ Anxiety ☐ Learning problems
- ☐ Short term memory problems
- ☐ Attention deficit disorder
- ☐ Other: ____

Gastrointestinal/Liver

- ☐ Chronic constipation ☐ Colon polyps
- ☐ Chronic diarrhea ☐ Intestinal disorder
- ☐ Ulcers ☐ Reflux/heartburn
- ☐ Gallstones ☐ Hepatitis
- ☐ Cirrhosis ☐ Pancreatic problems
- ☐ Other: ____

Genitourinary

- ☐ Bladder problems ☐ Chronic kidney failure
- ☐ Kidney stones
- ☐ Other: ____

Endocrine

- ☐ Diabetes mellitus ☐ Thyroid nodules
- ☐ Thyroid problems ☐ Pancreatic problems
- ☐ Growth hormone deficiency
- ☐ Vitamin D deficiency ☐ Diabetes insipidus
- ☐ Hyperprolactinemia
- ☐ Other: ____

Sexual/Reproductive

- ☐ Prostate problems ☐ Hysterectomy
- ☐ Sexual functioning problems ☐ Oophorectomy
- ☐ Problems with ovaries ☐ Vasectomy
- ☐ Problems with testicles ☐ Abnormal pap smears
- ☐ Problems with uterus
- ☐ Other: ____

Musculoskeletal

- ☐ Osteopenia/osteoporosis ☐ Joint pains
- ☐ Muscle problems ☐ Scoliosis
- ☐ Fractures ☐ Amputation
- ☐ Limb salvage ☐ Chronic pain
- ☐ Other: ____

Neurological

- ☐ Peripheral neuropathy ☐ Migraines
- ☐ Seizures ☐ Frequent headaches
- ☐ Other: ____

SURGERIES

Have you had any surgical procedures since the completion of therapy?

☐ Yes ☐ No

If yes, please list below:

1. Procedure: ______________________
   Month/Year: ______________________
2. Procedure: ______________________
   Month/Year: ______________________
3. Procedure: ______________________
   Month/Year: ______________________
4. Procedure: ______________________
   Month/Year: ______________________
5. Procedure: ______________________
   Month/Year: ______________________
6. Procedure: ______________________
   Month/Year: ______________________

NEW CANCERS

Have any new cancers developed since the completion of therapy?

☐ Yes ☐ No

If yes, please describe below:

First New Cancer

Diagnosis date (month/year): ______________________

Hospital/Clinic: ______________________

Diagnosis: ______________________

Tumor Site: ______________________

Treatment details: ______________________

Second New Cancer

Diagnosis date (month/year): ______________________

Hospital/Clinic: ______________________

Diagnosis: ______________________

Tumor Site: ______________________

Treatment details: ______________________

CURRENT MEDICATIONS

Include prescription and over-the-counter drugs, vitamins, etc.

1. ______________________
2. ______________________
3. ______________________
4. ______________________
5. ______________________
6. ______________________
7. ______________________
8. ______________________
9. ______________________
10. ______________________

MEDICATION ALLERGIES

Medication	Reaction

Thank you for completing the questionnaire!

Are there any additional issues/concerns would you like to discuss with the Long-Term Follow-Up health care team today?
